# Ranyard Nurses

**Published:** 1919-09-27

**Authors:** Irene M. Hett

**Affiliations:** Hon. Sec. The Ranyard Nurses, Ranyard House, 25 Russell Square, W.C.


### Ranyard Nurses.

*To the Editor of* THE HOSPITAL.

SIR,—May I comment on the paragraph in your issue of September 13, p. 598, with regard to the Ranyard Nurses. A district was opened on provident lines in connection with the Ranyard Mission as far back as 1903, and ever since then the system has been urged in areas where Ranyard Nurses are at work; but, unless those responsible in each district for raising the local funds will adopt the plan, it cannot be arranged from headquarters. From its earliest beginnings the Mission has taught self-help, and we are in entire sympathy with the method. It not only makes the work self-supporting, but enables the people to give small regular contributions, which they can generally afford, instead of having to raise a sum for nursing services at the time of sickness when financial difficulties are already great. With regard to the figure "under £25" quoted in your note, as patients' payments, perhaps I may be allowed to explain that this only refers to payments made for dressings, etc. In addition to this £25 a sum of £92 is shown on page 13 of our Report, which, moreover, only includes donations given by patients and sent direct to headquarters. In many cases such donations are given to the local secretary and are included in the subscriptions (pages 10-12) sent to headquarters from the local committee of the area where the nurse is at work. You will see from this that we are doing more than appears to obtain support from patients themselves, and a scheme is under consideration for increasing their contributions still further.—Yours faithfully,

IRENE M. HETT, Hon. Sec.
THE RANYARD NURSES,
Ranyard House, 25 Russell Square, W.C.

*September* 22, 1919.

[The main theme of Miss I. M. Hett's letter is finance. We suggest, therefore, that the finance statements should be consolidated so as to bring all receipts into one account, then the actual financial position each year can be clearly shown. This is a simple matter of entry and account, and might be adopted with advantage in the next report issued from the head office.—EDITOR THE HOSPITAL.]

